# Herbarium of Vascular Plants Collection of the University of Extremadura (Spain)

**DOI:** 10.3897/phytokeys.25.5341

**Published:** 2013-06-19

**Authors:** Marta Espinosa, Josefa López

**Affiliations:** 1 Área de Botánica, Facultad de Ciencias, Universidad de Extremadura, Avda. de Elvas s/n, 06006, Badajoz, Spain

**Keywords:** Extremadura, herbarium collection, Liliopsida, Magnoliopsida, Spain, Portugal, Baleares islands, Macaronesian region, Morocco, Brazil, UNEX Herbarium, vascular plant taxonomy

## Abstract

The herbarium of University of Extremadura (UNEX Herbarium) is formed by 36451 specimens of vascular plants whose main origin is the autonomous region of Extremadura (Spain) and Portugal, although it also contains a smaller number of specimens from different places, including the rest of peninsular Spain, the Baleares Islands, the Macaronesian region (Canary Islands, Madeira and Azores), northwest of Africa (Morocco) and Brazil. 98% of the total records are georeferenced.

It is an active collection in continuous growth. Its data can be accessed through the GBIF data portal at http://data.gbif.org/datasets/resource/255 and http://www.eweb.unex.es/eweb/botanica/herbario/. This paper describes the specimen associated data set of the UNEX Herbarium, with an objective to disseminate the data contained in a data set with potential users, and promote the multiple uses of the data.

## The UNEX Herbarium

Established in 1986, the specimens that the herbarium of vascular plants collection of University of Extremadura (UNEX Herbarium) incorporates is the result of the work of collections and identifications by different researchers, associated with the Botany Area (Department of Plant Biology, Ecology and Earth Sciences) of the University of Extremadura. Chief amongst these individuals include: Professor Juan Antonio Devesa, founder and director of the herbarium until 2004, and Dr. Trinidad Ruiz, curator and current director of the UNEX Herbarium. Significant contributions have also been made by Professor Ana Ortega-Olivencia, Dr. Rafael Tormo, Dr. Josefa López, and Dr. Tomás Rodríguez-Riaño. Other researchers who contributed to the growth of the UNEX Herbarium includes, Mª Carmen Viera, Jacinto Pedro Carrasco, Adolfo Muñoz, Inmaculada Montero, and Francisco Mª Vázquez. In addition, the disinterested work developed throughout time by a lot of students of the University of Extremadura, especially, Mª Luisa Navarro Pérez and Dr. Francisco Javier Valtueña.

The development of different research projects has made it possible to improve the quality of the herbarium. Among them, the numerous compilations carried out by differents members of the Botany Area in order to elaborate the first flora of the Extremadura region (Devesa 1995) or the synthesis of different taxonomic families or genera for Flora Ibérica (http://www.floraiberica.org/). Additionally, 4.5% of the material comes from the purchase of part of the herbarium of Dr. J.V.C. Malato-Beliz.

Limited and unpredictable funding has always pose challenge for ensuring sustained growth of the collection facility since its establishment.

## General taxonomic coverage description

**Figure 1.**
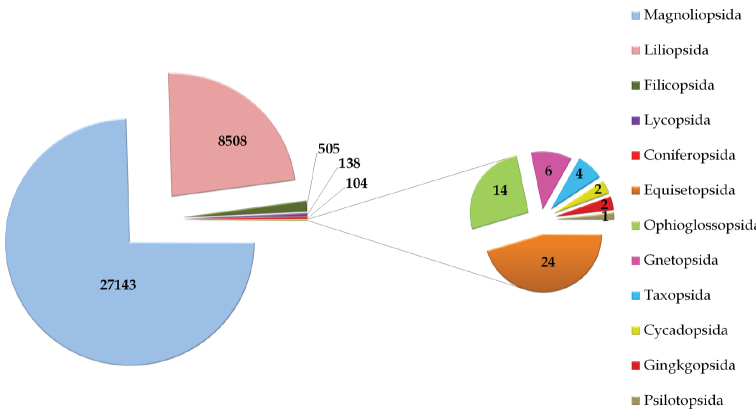
Taxonomic coverage (as per classes) of the UNEX Herbarium.

As depicted in Figure 1, majority of the specimens in UNEX Herbarium belong to class Magnoliopsida (27143 specimens) and Liliopsida (8508 specimens). These classes are followed by Filicopsida (505 specimens), Lycopsida (138 specimens), Coniferopsida (104 specimens), Equisetopsida (24 specimens), Ophioglossopsida (14 specimens), Gnetopsida (6 specimens), Taxopsida (4 specimens) Cycadopsida and Gingkgopsida (both with 2 specimens), and Psilotopsida (1 specimen).

UNEX herbarium represents 210 families, of which 22% and 21% of the specimens belongs to Poaceae and Fabaceae family respectively. This is followed by Asteraceae (15%), Scrophulariaceae (6%), Lamiaceae (6%), Caryophyllacae (5%), Brassicaceae (4%), Cyperaceae (4%), Rubiaceae (3%), Ranunculaceae (3%), Liliaceae (3%), Boraginaceae (3%), Apiaceae (3%), and Cistaceae (2%). The herbarium includes 1253 genera (Figure 3), significant ones amongst them are *Trifolium* (1345 specimens), *Ranunculus* (623 specimens), *Scrophularia* (583 specimens), *Vicia* (537 specimens), *Stipa* (505 specimens), *Galium* (479 specimens), *Juncus* (439 specimens), *Vulpia* (381 specimens), *Medicago* (365 specimens) and *Bromus* (342 specimens).

**Figure 2.**
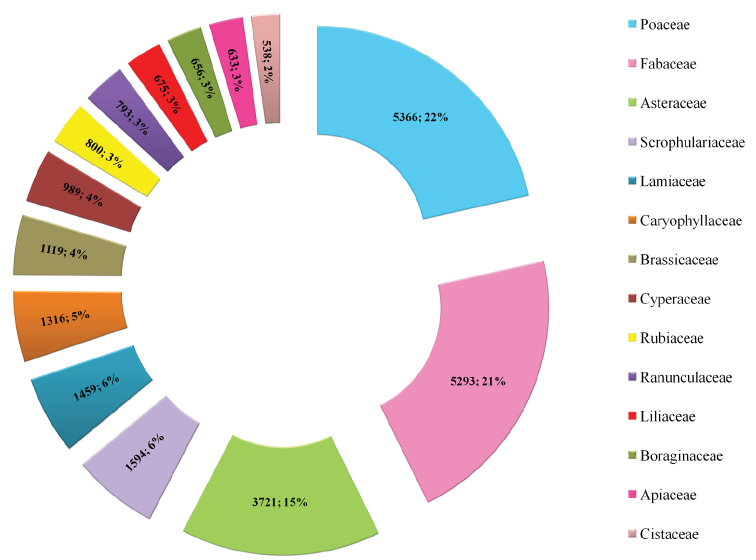
Taxonomic coverage (as per families) of the UNEX Herbarium.

**Figure 3.**
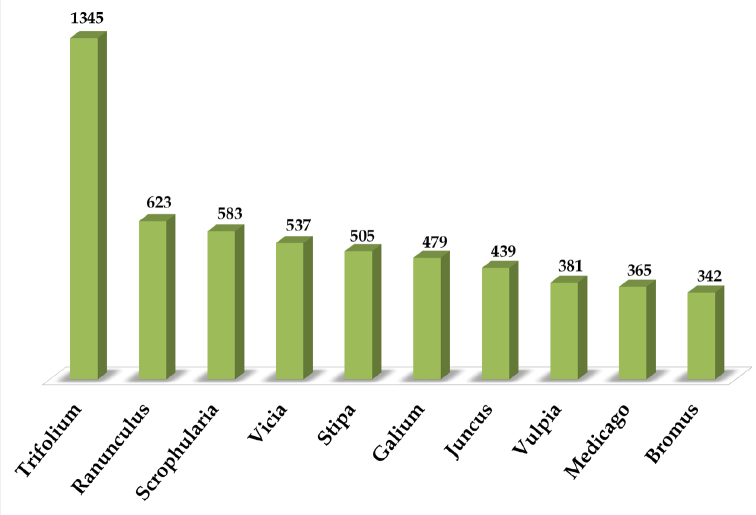
Taxonomic coverage (as per genus) of the UNEX Herbarium.

## Taxonomic ranks

**Kingdom:** Plantae

**Phylum:** Pteridophyta, Spermatophyta

**Class:** Magnoliopsida, Liliopsida, Filicopsida, Lycopsida, Coniferopsida, Equisetopsida, Ophioglossopsida, Gnetopsida, Taxopsida, Cycadopsida, Gingkgopsida, Psilotopsida.

**Family:** Acanthaceae, Aceraceae, Adiantaceae, Agavaceae, Aizoaceae, Alismataceae, Amaranthaceae, Amaryllidaceae, Anacardiaceae, Apiaceae, Apocynaceae, Aquifoliaceae, Araceae, Araliaceae, Araucariaceae, Arecaceae, Aristolochiaceae, Asclepiadaceae, Aspidiaceae, Aspleniaceae, Asteraceae, Athyriaceae, Azollaceae, Balanophoraceae, Balsaminaceae, Basellaceae, Begoniaceae, Berberidaceae, Betulaceae, Bignoniaceae, Blechnaceae, Boraginaceae, Brassicaceae, Buddlejaceae, Butomaceae, Buxaceae, Cactaceae, Callitrichaceae, Calycanthaceae, Calyceraceae, Campanulaceae, Cannabaceae, Cannaceae, Capparaceae, Caprifoliaceae, Caryophyllaceae, Casuarinaceae, Celastraceae, Ceratophyllaceae, Characeae, Chenopodiaceae, Cistaceae, Clethraceae, Clusiaceae, Cneoraceae, Commelinaceae, Convolvulaceae, Coriariaceae, Cornaceae, Crassulaceae, Cryptogrammaceae, Cucurbitaceae, Cunoniaceae, Cupressaceae, Cyathaceae, Cycadaceae, Cyperaceae, Davalliaceae, Dicksoniaceae, Dioscoreaceae, Dipsacaceae, Droseraceae, Ebenaceae, Elaeagnaceae, Elaphoglossaceae, Elatinaceae, Empetraceae, Ephedraceae, Equisetaceae, Ericaceae, Euphorbiaceae, Fabaceae, Fagaceae, Flacourtiaceae, Fontinalaceae, Frankeniaceae, Fumariaceae, Gentianaceae, Geraniaceae, Gesneriaceae, Ginkgoaceae, Globulariaceae, Grossulariaceae, Guttiferaceae, Haloragaceae, Hamamelidaceae, Hemionitidaceae, Hippocastanaceae, Hydrangeaceae, Hydrophyllaceae, Hymenophyllaceae, Hypolepidaceae, Iridaceae, Isoetaceae, Juglandaceae, Juncaceae, Juncaginaceae, Lamiaceae, Lauraceae, Lemnaceae, Lentibulariaceae, Liliaceae, Linaceae, Loranthaceae, Lycopodiaceae, Lythraceae, Magnoliaceae, Malpighiaceae, Malvaceae, Maranthaceae, Marsileaceae, Melastomataceae, Meliaceae, Melianthaceae, Mimosaceae, Molluginaceae, Monimiaceae, Moraceae, Myoporaceae, Myricaceae, Myrsinaceae, Myrtaceae, Najadaceae, Nyctaginaceae, Nymphaeaceae, Oleaceae, Onagraceae, Ophioglossaceae, Orchidaceae, Orobanchaceae, Osmundaceae, Oxalidaceae, Paeoniaceae, Papaveraceae, Passifloraceae, Phytolaccaceae, Pinaceae, Piperaceae, Pittosporaceae, Plantaginaceae, Platanaceae, Plumbaginaceae, Poaceae, Podocarpaceae, Polemoniaceae, Polygalaceae, Polygonaceae, Polypodiaceae, Pontederiaceae, Portulacaceae, Potamogetonaceae, Primulaceae, Proteaceae, Psilotaceae, Pteridaceae, Punicaceae, Pyrolaceae, Rafflesiaceae, Ranunculaceae, Resedaceae, Rhamnaceae, Rosaceae, Rubiaceae, Ruppiaceae, Rutaceae, Salicaceae, Santalaceae, Sapindaceae, Saxifragaceae, Scrophulariaceae, Selaginellaceae, Simaroubaceae, Sinopteridaceae, Solanaceae, Sparganiaceae, Sterculiaceae, Styracaceae, Symplocaceae, Tamaricaceae, Taxaceae, Theaceae, Theligonaceae, Thelypteridaceae, Thymelaeaceae, Tiliaceae, Tropaeolaceae, Typhaceae, Ulmaceae, Umbelliferaceae, Urticaceae, Valerianaceae, Verbenaceae, Violaceae, Vitaceae, Zannichelliaceae, Zingiberaceae, Zygophyllaceae

## General spatial coverage

**Figure 4.**
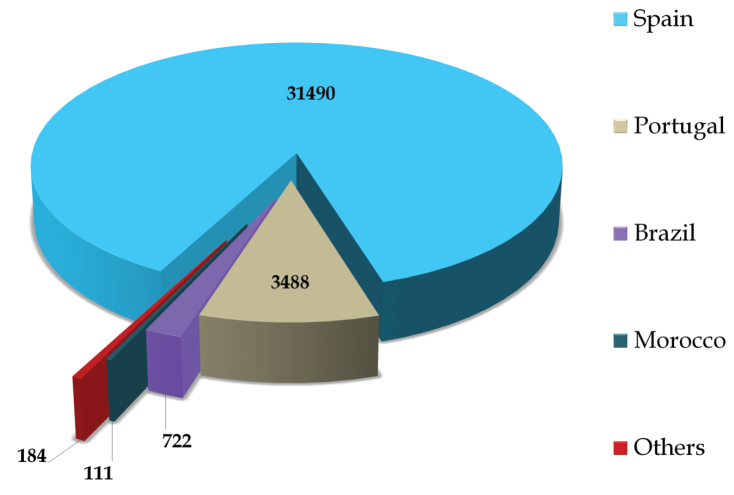
Geographic coverage of the UNEX Herbarium.

Specimens deposited in the UNEX Herbarium have been collected mainly from Iberian Peninsula (Spain and Portugal) northwest of Africa (Morocco) and Brazil. As indicated in Figure 4, maximum number of specimens included in the dataset are collected from Spain (31490) followed by Portugal (3488), Brazil (722) and Morocco (111) respectively. With regards to collections from Spanish provinces, Badajoz contributes 16910 specimens, followed by Cáceres (6855 specimens) and Cádiz (1012 specimens). Other sampling areas include Almería (711 specimens), Jaén (495 specimens), Málaga (411 specimens), Gerona (388 specimens), Huesca (371 specimens), León (308 specimens), Oviedo (293 specimens), Granada (290 specimens), Huelva (260 specimens) and Lérida (249 specimens).

The specimens better represented in the database correspond to elements of the Mediterranean forest and/or scrubland. These specimens are developed under a seasonal climate of mild winters and hot and dry summers, with abundant rainfall in autumn and spring. Frequent in this climate are forest fires, to which the vegetation is adapted. Specimens from Brazil (except one specimen collected in the state of Santa Catarina), are collected in the state of Río Grande do Sul. This state has a humid subtropical climate and a hydrography classified into three major regions: Uruguay River Basin, Guaiba River, and Litoral.

**Coordinates:** 38°0'0"S and 52°0'0"N Latitude; 115°0'0"W and 65°0'0"E longitude.

## Temporal coverage

**Figure 5.**
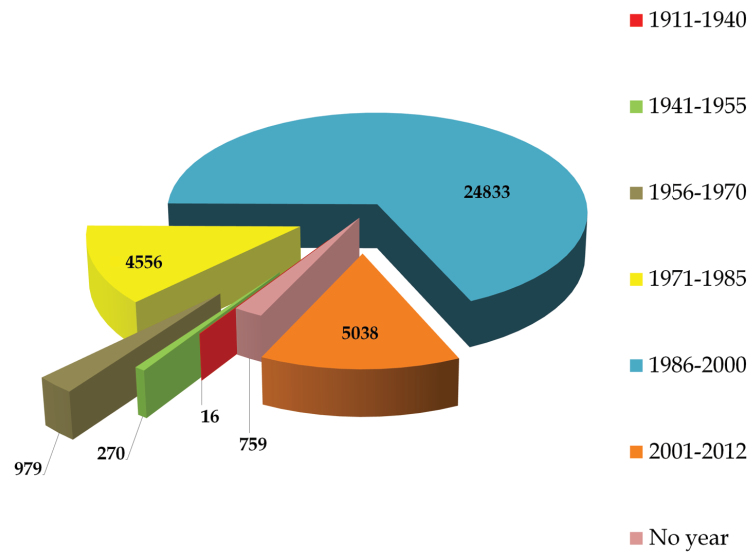
Temporal coverage of the UNEX Herbarium.

1911 – 2013. As shown in Figure 5, earliest collection event is dated back to 1911. Maximum number of specimens are collected during 1986–2000 (24833), followed by 2001–2012 (5038), 1971–1985 (4556), 1956–1970 (979), 1941–1955 (270), and 1911–1940 (16). There are 759 specimens for which period of collection cannot be ascertained.

**Collection name:** UNEX Herbarium, University of Extremadura.

**Collection identifier:** http://data.gbif.org/datasets/resource/255

**Formation period:** 1986-2013

**Specimen preservation method:** Dried and pressed

**Curatorial unit:** 36451 with an uncertainty of 0 (Sheets)

**Curatorial unit:** 1253 with an uncertainty of 0 (Genera)

## Methods

### Method step description

Dried and pressed method has been adopted for preservation of the specimens deposited in the collection. After collection of specimens in the field (see “sampling description” for the collection protocol) they are pressed and dried with the aim of adapting them to a flat surface and remove water from the tissue, thereby preventing their degeneration or attacks by bacteria, fungi or insects that would destroy them.

Assembly of specimens: The dried material is mounted on a holder constituted by an A3 cardboard (42 × 29.7 cm) and a resistant paper (so-called jacket) dimension A2 (42 × 59.4 cm) that perfectly covers the cardboard, thus protecting the specimen. The assembly is made on the cardboard with transparent tape, allowing both that the specimen be tighten to the holder and that the assembly be aesthetic.

Registration of herbarium specimens: After assembly, specimens are registered and labeled. Registration is done in a database (Microsoft Office Access Database → DarwinCore 1.2) in which each specimen is assigned a reference number allocated consecutively. The information contained in the record of each specimen is: institution owner of the herbarium, reference number, scientific name of the family and species, date of collection, georeferencing data (country, province, town), habitat, Legitimavit, and Determinavit. All log data are printed and constitute the sheet label.

Treatment of specimens: Before putting the specimens in the herbarium they are stored in hermetic plastic boxes and kept for 72 hours in cold storage (freezers) at -40°C. In this way the material is decontaminated from possible attacks of pathogens that can destroy them and the rest of material already in the herbarium.

Storage of specimens: Finally, the specimens are kept inside compact enclosures in shelves where they are arranged taking into account the four main groups: pteridophytes, gymnosperms, monocots, and dicots. Within each main group the specimens are alphabetical arranged by families and genera.

### Study extent description

**Figure 6.**
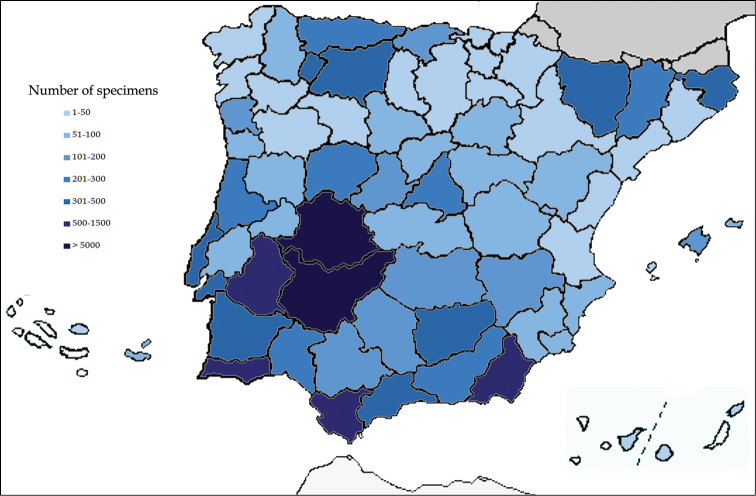
Geographical distribution of specimens in the Iberian Peninsula, Baleares, Canarias, Madeira and Azores.

**Figure 7.**
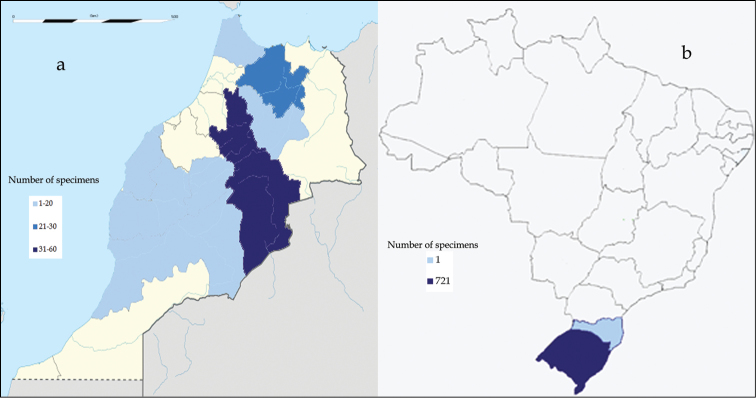
Geographical distribution of specimens in (**a**) Morocco and (**b**) Brazil.

Iberian Peninsula is the most significant geographic zone represented in UNEX Herbarium. Figure 6 depicts the collections from various provinces of Iberian Peninsula. Over 5000 specimens are collected from two provinces (Badajoz and Cáceres). Two provinces (Cádiz and Almería) contributed specimens in the range of 500-1500. The specimens collected from morocco (111 sheets) are representatives for an area greater than 50% of the country (Fig. 7a). Conversely, specimens from Brazil (721 sheets) cover not more than 3% of the area of the country (Fig. 7b).

### Sampling description

As evident from the previous section, the specimens deposited in the UNEX Herbarium comes from diverse regions, which is outcome of several research projects depositing the specimens. As a result, specimens are not collected using a single, uniform protocol. Of the materials from donations or purchases (e.g. herbarium of Dr. J.V.C. Malato-Beliz) the protocol followed for the collection of specimens is unknown. The methodology used in collecting plants by researchers from the Botany Area may change depending on the objectives pursued by the work they are carried out for.

In general, collection takes place following previous available references drawn from scientific works, herbarium material or indications from reliable collectors. The collection campaigns are designed to be more or less exhaustive of different available areas depending on the purpose of the collection (e.g. for taxonomic works, we do exhaustive inspections of whole territories with expansion of the collection area beyond known indications).

### Quality control description

Each specimens and associated data record was subjected to two quality procedures; (a) taxonomic determination or identification and (b) geo-referencing. For taxonomic identification, trusted experts were contacted, the 85% of the material has been identified by investigators of the Botany Area of the University of Extremadura. These experts have authored number of monographs, which include Vegetación y Flora de Extremadura (Devesa 1995) in which 2050 taxa’s are described. Other includes Las Gramíneas de Extremadura (Devesa 1999) or the volumes of Flora iberica XV (Devesa et al. 2007) comprising the taxonomic synthesis of families Adoxaceae, Caprifoliaceae, Dipsacaceae, Rubiaceae and Valerianaceae, and XVI (under edition, see http://www.floraiberica.org/) in which these experts are responsible for the genera *Arctium*, *Atractylis*, *Carlina*, *Crupina*, *Cynara*, *Echinops*, *Onopordum*, *Rhaponticum*, *Saussurea*, *Staehelina*, and *Xeranthemum*. It is worth also to note the publications of new species of flora (*Centaurea bethurica*, Devesa and López 2008; *Scrophularia fontqueri* Ortega-Olivencia and Devesa 1998; *Galium moralesianum* and *Galium talaveranum* Ortega-Olivencia and Devesa 2003; *Galium belizianum* Ortega-Olivencia et al. 2004), whose *typus* are preserved in UNEX Herbarium.

Besides the above mentioned authoritative literature, experts have also relied upon the biology of the reproduction of different taxa of the family Fabaceae (López et al. 1998, 1999a, 1999b, 2000, Ortega-Olivencia and Devesa 1997, Ortega-Olivencia et al. 1997, 2005, Rodríguez-Riaño et al. 1999a, 1999b, 1999c, 2004, 2006, Valtueña et al. 2007, 2008a, 2008b, 2010a, 2010b, 2011, 2012) or the genera *Drosophyllum* (Ortega-Olivencia et al. 1995, 1998) or *Scrophularia* (Ortega-Olivencia and Devesa 1993a, 1993b, Ortega-Olivencia et al. 2012, Valtueña et al. 2013). Use of these literature resources and long standing experience of the researchers high degree of confidence to the taxonomic identification of the specimens.

The 98% of the records in the collection are georeferenced. A total of 58.38% have MGRS coordinates and the rest geographical coordinates. The MGRS coordinate system has been transformed into geographical coordinates through a geographic calculator (http://www.asturnatura.com/sinflac/calculadora-conversiones-coordenadas.php), while at the same time maintaining the MGRS coordinates in the database. The accuracy of these coordinates grids varies from 1 km^2^ to 10 km^2^. The geographical coordinates have been taken through the description of localities and search of these localities in Google Earth (http://www.google.com/earth/index.html). The accuracy of geographic coordinate values ​​also varies between 2 and 12 km depending on the number of decimal places contained.

## Datasets

**Object name:** Herbarium of Vascular Plants Collection of the University of Extremadura (Spain)

**Character encoding:** UTF-8

**Format name:** Darwin Core Archive format

**Format version:** 1.0

**Distribution**

http://www.gbif.es:8080/ipt/resource.do?r=collectionherbariumextremadura

**Publication date of data:** 2013-05-18

**Language:** English

**Licenses of use:** This database “Herbarium of Vascular Plants Collection of the University of Extremadura (Spain)”is made available under license Open Data Commons Attribution: http://www.opendatacommons.org/licenses/by/1.0/

**DarwinCore elements:** Twenty two (22) DarwinCore (http://purl.org/dc/terms/) elements included in the dataset published through the GBIF network. These are (a) modified, (b) infraspecificEpithet, (c) eventDate, (d) family, (e) basisOfRecord, (f) kingdom, (g) typeStatus, (h) collectionCode, (i) catalogNumber, (j) scientificName, (k) locality, (l) individualCount, (m) scientificNameAuthorship, (n) institutionCode (o) decimalLongitude, (p) country, (q) preparations, (r) identifiedBy, (s) stateProvince, (t) recordedBy, (u) recordNumber, (v) decimalLatitude, (w) genus, (x) specificEpithet and (y) occurrenceRemarks.

**Character encoding:** iso-8859-1

**Format name:** Access

**Format version:** 1.0

**Distribution:** http://data.gbif.org/datasets/resource/255

**Metadata language:** English

**Date of metadata creation:** 2013-03-20

**Hierarchy level:** Dataset
